# Progress on Applying Carbon Dots for Inhibition of RNA Virus Infection

**DOI:** 10.7150/ntno.73918

**Published:** 2022-08-21

**Authors:** Yaung Kwee, Yiqun Zhou, Mochamad Zakki Fahmi, Madhuri Sharon, Alfinda Novi Kristanti

**Affiliations:** 1 Department of Chemistry, Pakokku University, Myaing Road, Pakokku 90401, Myanmar.; 2 Department of Chemistry, University of Miami, Coral Gables, FL 33146, USA.; 3 Department of Chemistry, Universitas Airlangga, Surabaya 60115, Indonesia.; 4 Supramodification Nano-micro Engineering Research Group, Universitas Airlangga, Surabaya 60115, Indonesia.; 5 Research Director at Walchand Center for Research in Nanotechnology and Bionanotechnology, Walchand College of Arts and Science, W. H. Road, Ashok Chowk, Solapur 413006, India.

**Keywords:** RNA virus, virus infection treatment, carbon dots, nanomedicine, antiviral therapy

## Abstract

Viral infection is a globally leading health issue. Annually, new lethal RNA viruses unexpectedly emerged and mutated threatening health and safety. Meanwhile, it is urgent to explore novel antiviral agents, which, however, takes years to be clinically available. Nonetheless, the development of carbon dots (CDs) in the past 20 years has exhibited their vast application potentials and revealed their promising capacity as future antiviral agents considering their versatile properties and significant antiviral responses. Thus, CDs have been widely investigated as an alternative of traditional chemotherapy for inhibiting viral infection and replication *in vitro*. Meanwhile, attempts to apply CDs to *in vivo* systems are in high demand. In this review, recent developments of CDs-based antiviral therapies are systematically summarized. Furthermore, the role of CDs in photodynamic inactivation to kill viruses or bacteria is briefly discussed.

## Introduction

An RNA virus indicates a virus that has RNA as its genetic material. Notable human diseases that result from RNA virus infection include the common cold, influenza, SARS, COVID-19, MERS, hepatitis C, E, West Nile fever, Ebola virus disease, rabies, mumps and acquired immune deficiency syndrome (AIDS) 1. Although the genetic material of a virus can be either DNA or RNA, RNA viruses generally have higher mutation rates since viral RNA polymerases lack the proofreading ability as DNA polymerases 2, which explains why it is difficult to create effective vaccines against RNA viruses 3. As a typical RNA virus model, since the 1980s, the human immunodeficiency virus (HIV) has infected 33 million people all over the world. Meanwhile, there have been more than 3 million new cases and 2 million deaths each year ever since 4. And the COVID-19 since its break out in 2019, globally, as of 6:34 pm CEST, 28 July 2022, there have been 571,198,904 confirmed cases of COVID-19, including 6,387,863 deaths, reported to WHO. Thus, it is very urgent and significant to solve these health problems induced by various RNA virus infections.

In terms of antiviral therapy, currently, drug delivery systems (DDS) combined with highly active antiretroviral therapy (HAART) is a common practice. It increases the longevity of HIV-infected patients. However, some challenges still remain 5.

Despite the great advances in technology for virus management, viruses cannot be eradicated by current treatment of HAART due to considerable restrictions and the main challenge during the course of treatment is poor patient compliance in the long run, which usually leads to a poor therapeutic efficacy and requires a longer treatment period 6. Hence, an alternative nanotechnological approach has timely emerged as a scientifically potential solution in a short-term pursuit of an effective therapeutic strategy against HIV and other RNA viruses, such as dengue virus (DENV), Ebola virus (EBOV), and pulmonary syndrome corona 7, 8.

Nanotechnology has tremendous potential to fight against viruses with nanoparticles (NPs) 9. Those nanoscale particles are able to penetrate small tissue systems and play as prominent nanomedicine or drug nanocarriers for the treatment of various viral diseases and cancers 10. The antiviral activity of NPs has been standardized through various mechanisms to provide their antiviral mode of action: small particle size, high surface areas to volume ratios, tuneable surface electric charge, which will benefit their drug delivery, drug loading, and cell penetration, respectively 11-13.

Additionally, encapsulation, surface functionalization, and structural modifications can optimize drug dosing, which enhances delivery towards anatomically privileged cellular sites, specific targeted tissues, or subcellular compartments 14. Moreover, nanotechnology platforms for diagnosis of RNA viruses consist of polymer NPs 15, liposomes 16, dendrimers 17, nano-emulsions 18, and nanosuspensions 19. These nanomaterials have been applied to make considerable efforts to fight viral threats to some limited level but their water solubility, patient adherence, biostability, and bioavailability need to be further improved 20.

Furthermore, NPs have been synthesized for HIV and other RNA viruses' inhibition such as noble metal-attributed drug-delivery agents 21, polymers 22, composite materials 23, and lipids 24. However, uses of these NPs are limited by their toxicity, inert properties of starting material, colloidal instability, and complicated synthesis procedures 25. Therefore, many nanomaterials have been developed, such as carbon dots (CDs), to address all the aforementioned issues while diminishing these health problems around the world.

CDs have exhibited excellent antiviral activities due to some aforementioned properties and high affinity of multifunctional groups to various specific cellular sites 26. For instance, the outer shell of HIV virus is composed of two layers of lipids with multiple proteins spikes including gp120 and gp41 glycoproteins 27. The binding between HIV and T-cells is due to the interaction between gp120 and CD4 receptors on T cells. CDs with certain surface functional moieties attract those spike proteins of gp120 receptors, and thus CDs can be bound to HIV. Consequently, the use of CDs will inhibit viral entry into host cells. There are some literature review articles in terms of nanotechnology-approach-derived nanomedicine for RNA viruses' theranostics 28-33. Apart from these strategies, the current review focuses on the nanotechnology where CDs were shown as powerful antiviral agents against RNA viruses. Eventually, the current review aims to largely outline the potential of CDs in enhanced virus prevention and treatment.

## Syntheses of Carbon Dots

CDs, a scientifically interesting class of carbon-based nanomaterial with a size below 10 nm, were unexpectedly discovered when single-walled carbon nanotubes (SWCNTs) were purified through electrophoresis method in 2004 34, 35. In 2006, Sun et al. prepared CDs from finely ground graphite and cement dust particles through laser ablation 36. The synthetic approaches of CDs generally include top-down and bottom-up approaches. The top-down approach covers acid dehydration, laser ablation of graphite, laser passivation, plasma treatment, electrochemical or chemical oxidation, and arc discharge on diverse carbon-based starting materials. On the contrary, the bottom-up approach includes pyrolysis, thermal carbonization, microwave or ultrasonic treatments, and hydrothermal/solvothermal treatments on various small organic molecules or compounds 37, 38. Biogenic synthesis of CD using of biological entities as precursors such as microbes, fungi, plant extracts, plant metabolites and secretes, and also metabolites of the animal; have become more prevalent for its use in biosystem.

## Properties of Carbon Dots

CDs exhibit many outstanding merits including excellent water solubility/dispersity, chemical inertness, good biocompatibility, low toxicity, cost‐effective synthesis, good elasticity for surface modifications, photoluminescence (PL) and good photostability against photo-bleaching 39. They are extensively applied in the fields of bio‐imaging enhancement 40, bio‐sensing 41, photodynamic therapy 42 photocatalysis 43, thermoelectricity 44, hybrid rocket fuel 45, drug-delivery 46, antimicrobial 47, 48, and virus inhibition 49. The PL mainstream mechanism of CDs is ascribed to quantum confinement effect, surface state and molecular state. The fluorescence intensity could be varied with respect to the emission turned from ultraviolet to near infrared (NIR). The fluorescence emission of CDs in the NIR wavelength range is effective for deep tissue penetration 50. In addition, CDs' surfaces are functionalized by miniature organic compounds, metals, and polymers so that their water dispersibility and nano fluorescence were enhanced 51. Furthermore, currently, nanoscale functional materials have emerged as novel antiviral agents because of their unique chemical and physical properties. In a nutshell novel and unique properties of CD that have given impetus to its use in theranostics are its's crystallinity, emission property, and electrical conductivity. However, little information is available about the potential effects of CDs on viruses.

## Mechanisms of Antiviral Action of Carbon Dots

Theoretically speaking, the spike proteins on viruses interact with cell membrane receptor proteins to enter host cells via endocytosis and rapidly replicate inside host cells. Therefore, the receptor proteins on host cells and the viruses' spike proteins are the main objects to target in the regulation of viral entry. Meanwhile, based on literature records, CDs play key roles in combating viral entry and replication with different mechanisms at different steps of viral infection that include attachment, penetration, replication, and budding. Recent efforts have shown that CD derived from biogenic sources like curcumin, glycyrrhizin, etc. are more promising because of their biocompatibility. Moreover, surface passivation/functionalization of CDs with positive charge and more hydrophilic groups on surface will not only help in understanding the mechanism of antiviral activity of CD, it will also enhance the anti-viral action, reduce the toxicity, so as to go ahead with *in vitro* as well as *in vivo* antiviral activity.

### Viral Inhibition at Attachment and Penetration Stages

The primary step of virus infection is viral attachment to the host cell, thus hindrance to this step will inactivate the virus. The mechanism to inhibit viral attachment involves suppression of the binding of gp120 on HIV viruses to CD4, a receptor protein, and co-receptors (CCR5 or CXCR4) on T cells, thereby blocking the viral entry process. However, suppressing the binding between viruses and host cells has a weakness that might lead to failure of disinfection when NPs disengage from the surfaces of either viruses or host cells 52.

**Figure 2:**
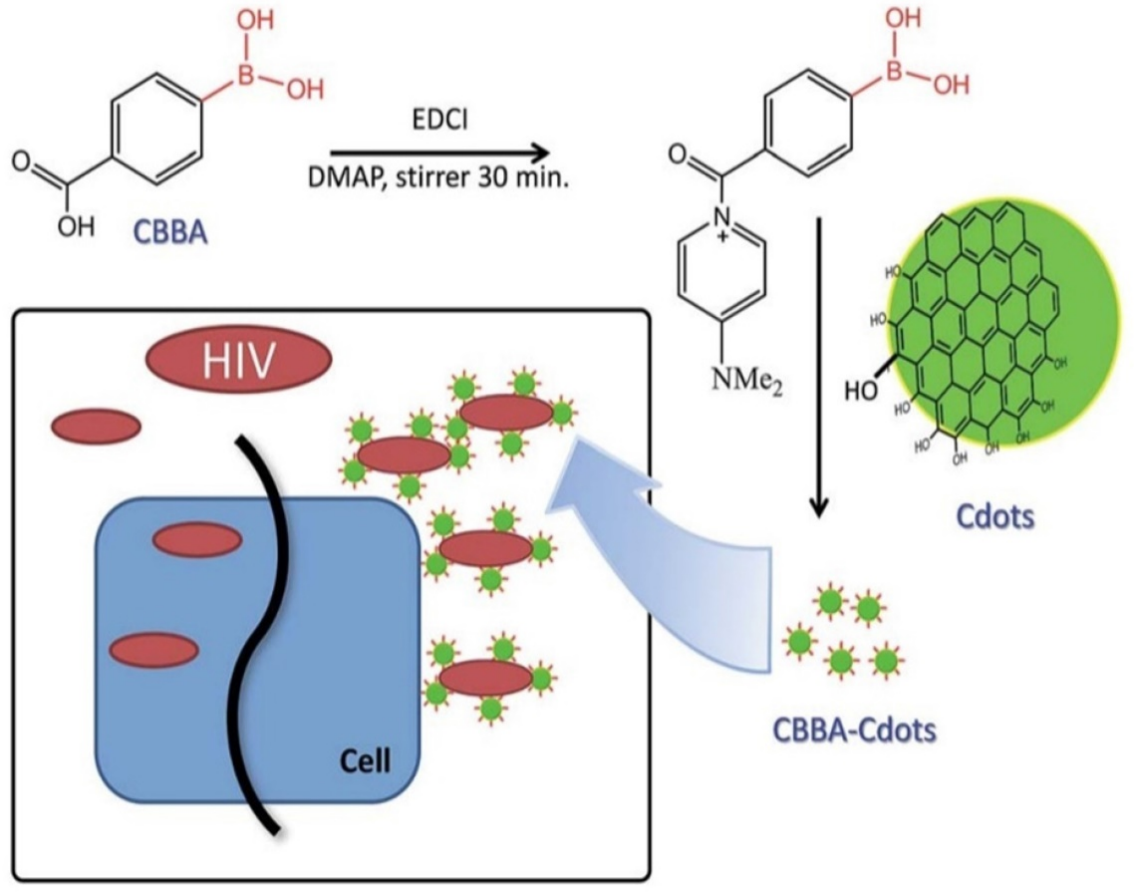
A graphitic illustration of conjugating CBBA to CDs and mechanism of viral entry inhibition. Reprinted with the permission of the reference 26.

Fahmi and co-workers applied CDs as a viral entry inhibitor to prevent attachment of gp 120 of HIV viruses to CD4 or co-receptor CCR5 on host cells (Figure 2). 26 The CDs were synthesized with citric acid (CA) as a sole precursor via pyrolysis. A graphene-like structure was observed on the CDs with abundant functional groups such as -OH, -COOH on the edge. To enhance virus inhibition, the prepared CDs were conjugated with carboxyl phenylboronic acid (CBBA). In this case, the boronic acid-modified CDs could bind to viral spikes and thus improve blockage of viral entry. In comparison, the antiviral activity of CBBA-modified CDs displayed an enhancement in inhibiting HIV infection than unmodified CDs. As to their cytotoxicity assessments, the average percentage of cell viabilities at different concentrations of CDs and CBBA-CDs were higher than 80%, revealing both are non-toxic to human cells. Their findings highlighted that the intrinsically antiviral property of boronic acid-modified CDs could be used in HIV prevention and treatment 26.

**Figure 3:**
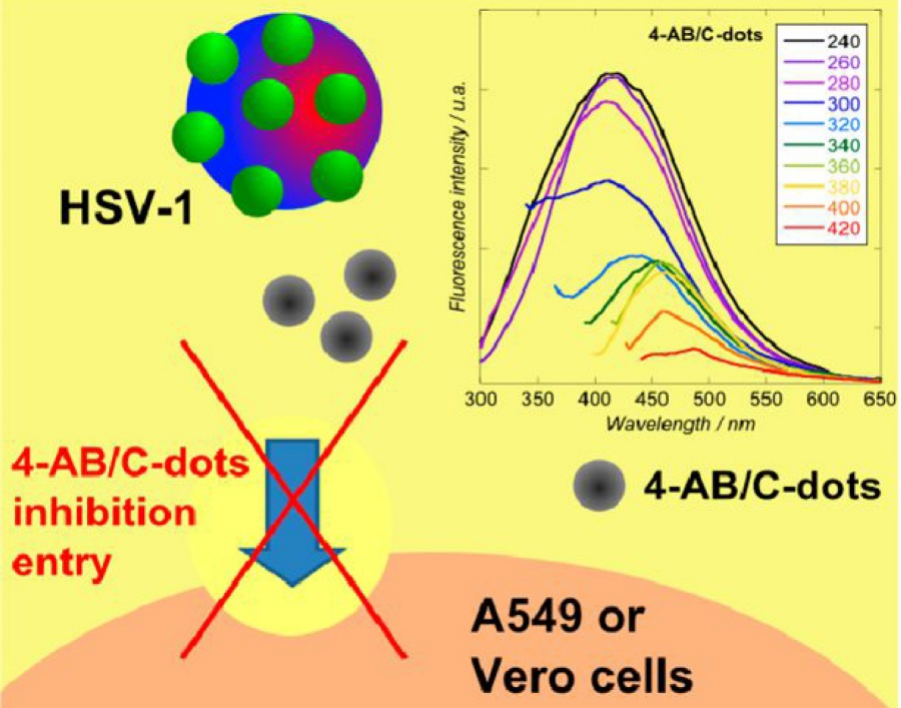
Mechanism of inhibition of viral entry by 4-aminophenylboronic acid hydrochloride derived CDs. Reprinted with permission from the reference 53.

Moreover, Barras et al. 53 investigated the potential of surface-functionalized CDs with boronic acid or amine to prohibit the viral entry of herpes simplex virus type 1 (HSV-1) as depicted in Figure 3. They implied that abundant ligands on the edge of nanostructure of CDs enhanced affinities toward targeted receptors. It was found that the prepared CDs inhibited HSV-1 infection in the concentration range of nanograms per millilitre (EC50 = 80 and 145 ng mL^-1^ on Vero and A549 cells, respectively) acting on the early stage of viral entry process through an interaction between CDs and the viruses and probably the cells at the same time 53.

**Figure 4:**
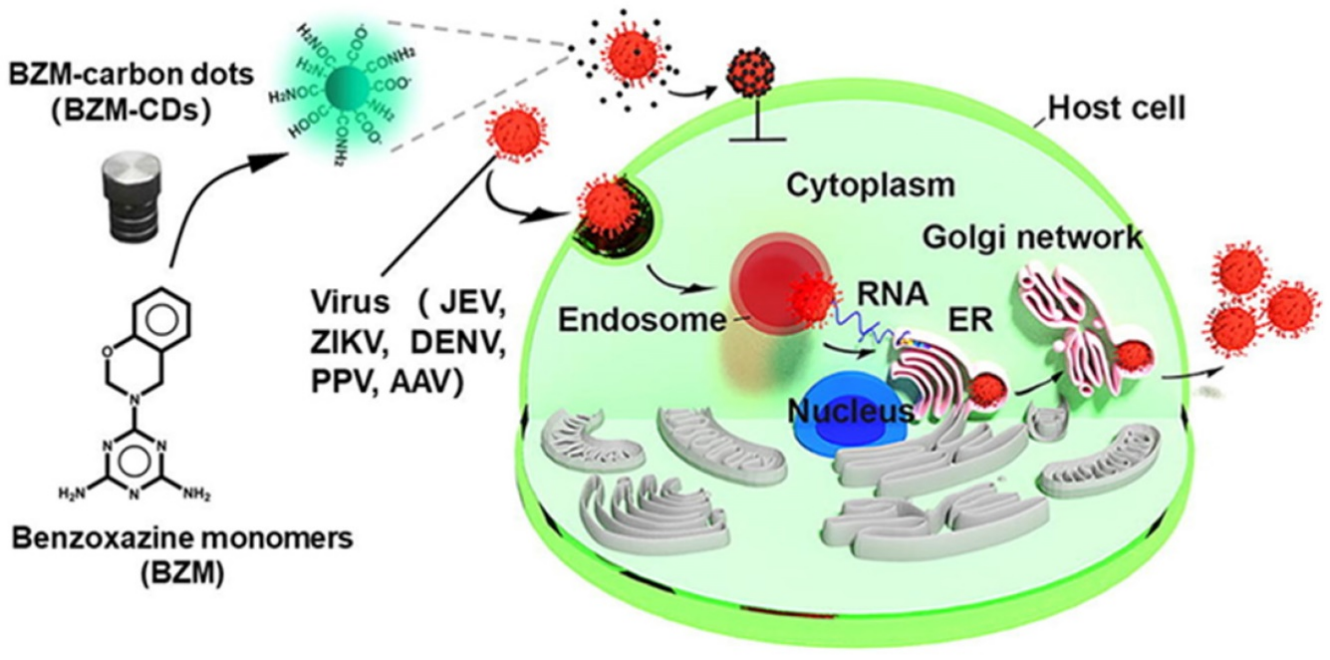
The graphical abstract framework of the prepared BZM-CDs to block viral infectivity. Reproduced with the permission from the reference 54.

Another study reported by Huang et al. in 2019 was related to the CDs (BZM-CDs) synthesized from a series of benzoxazine monomer. BZM-CDs showed their virus-blocking activity in case of death-dealing with flaviviruses (Japanese encephalitis, dengue viruses, and Zika) and non-enveloped viruses (porcine parvovirus and adenovirus-correlated virus) *in vitro* as described in Figure 4. It was observed that BZM-CDs immediately adhered to the virion surface, and the initiative of virus-cell interconnection was destroyed by those CDs. It was confirmed that the BZM-CDs could contribute to a fascinating broad-spectrum technique to suppress viral infections 54.

**Figure 5:**
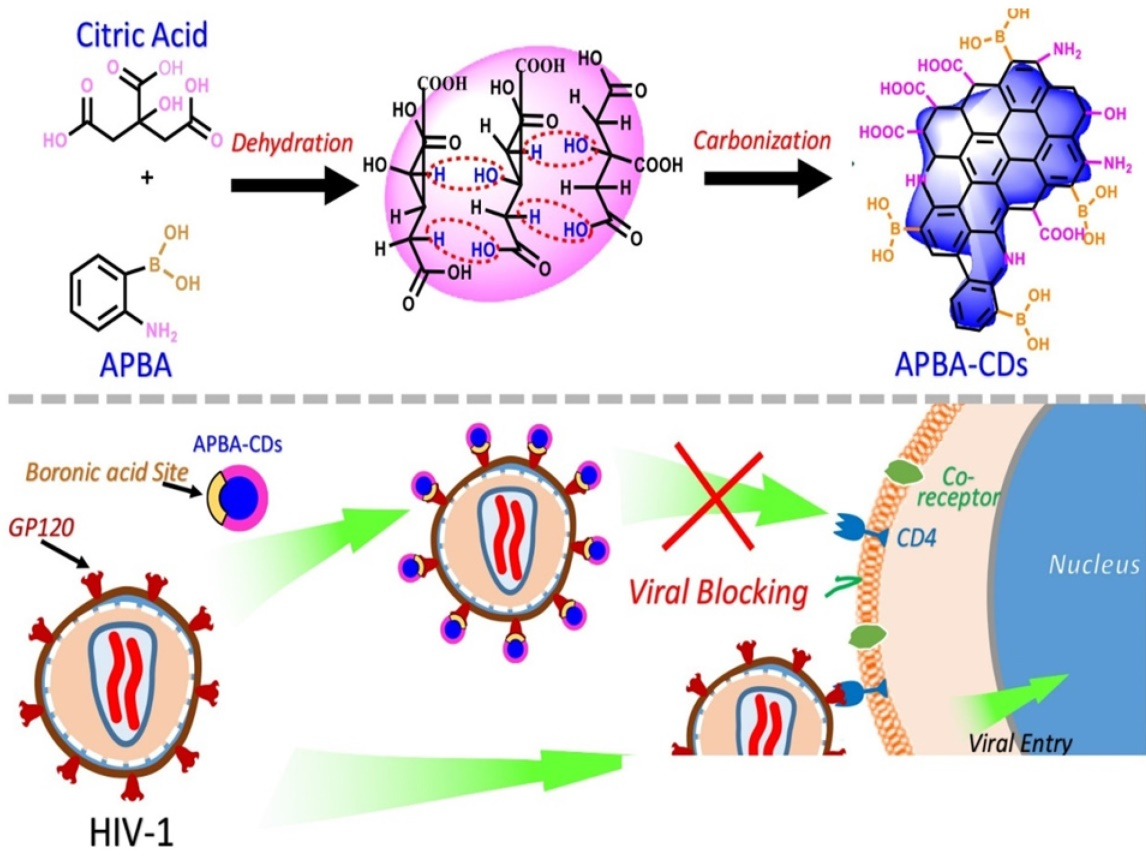
The schematic representation of the synthetic process of APBA-CDs via carbonization and function of APBA-CDs in blocking HIV-1 infection to MOLT-4 cells. Reprinted with permission from the reference 55.

Moreover, based on the aforementioned study, Aung et al. in 2020 fabricated amino phenylboronic acid-modified CDs (APBA-CDs) and employed the newly modified CDs as a novel antiviral agent targeting gp 120 and inhibiting HIV-1 viral entry process (Figure 5) 55. Their finding demonstrated that in terms of the mechanism of antiviral action, boronic acid-modified APBA-CDs more likely interacted with HIV-1 instead of host cells. It was presumed that the interaction between gp 120 and boronic acid strengthened the attachment of HIV-1 to CDs. In this regard, the important role of boronic acid was highlighted for blocking the viral entry of HIV-1. In addition, the boronic acid on APBA-CDs possibly formed chemical bonding with gp120, which could further disrupt viral contagion. Those boronic acid-modified CDs attracted amino moieties of reverse transcriptase containing HIV and prevented the reverse transcriptase from taking effect in the viral life cycle. Those APBA-CDs showed remarkable biocompatibility, low cytotoxicity (the CC_50_ reached up to 11.2 mg mL^-1^) and an excellent potential in inhibiting HIV-1 entry into specifically targeted cells 55.

### Virus Inhibition by Ceasing Replication Stage

Once the virus enters the host cell, strategies for interference include prevention of viral replication or budding. Inhibition of viral replication can be achieved by changing enzymes which are required for viral genome replication.

The role of triazole functionalized heteroatom co-doped carbon dots against human coronaviruses i.e. SARS-CoV-2 could be explored. Functionalization of nanomaterials is an important principle for highly anticipated outcomes to producing antiviral agents through both non-covalent bonding (hydrophobic interactions, electrostatic interactions, and π-π stacking) and covalent functionalization strategies. For example, the in-progress potential of triazole-based CDs template was proposed using a series of bioisosteres as antiviral agents to treat human coronavirus infection. CDs have a lot of hydrophilic functional groups on the edges that make them suitable for diverse biomedical applications. The surface functionality of CDs is vital to tune the level of interactions with the viruses 56.

**Figure 6:**
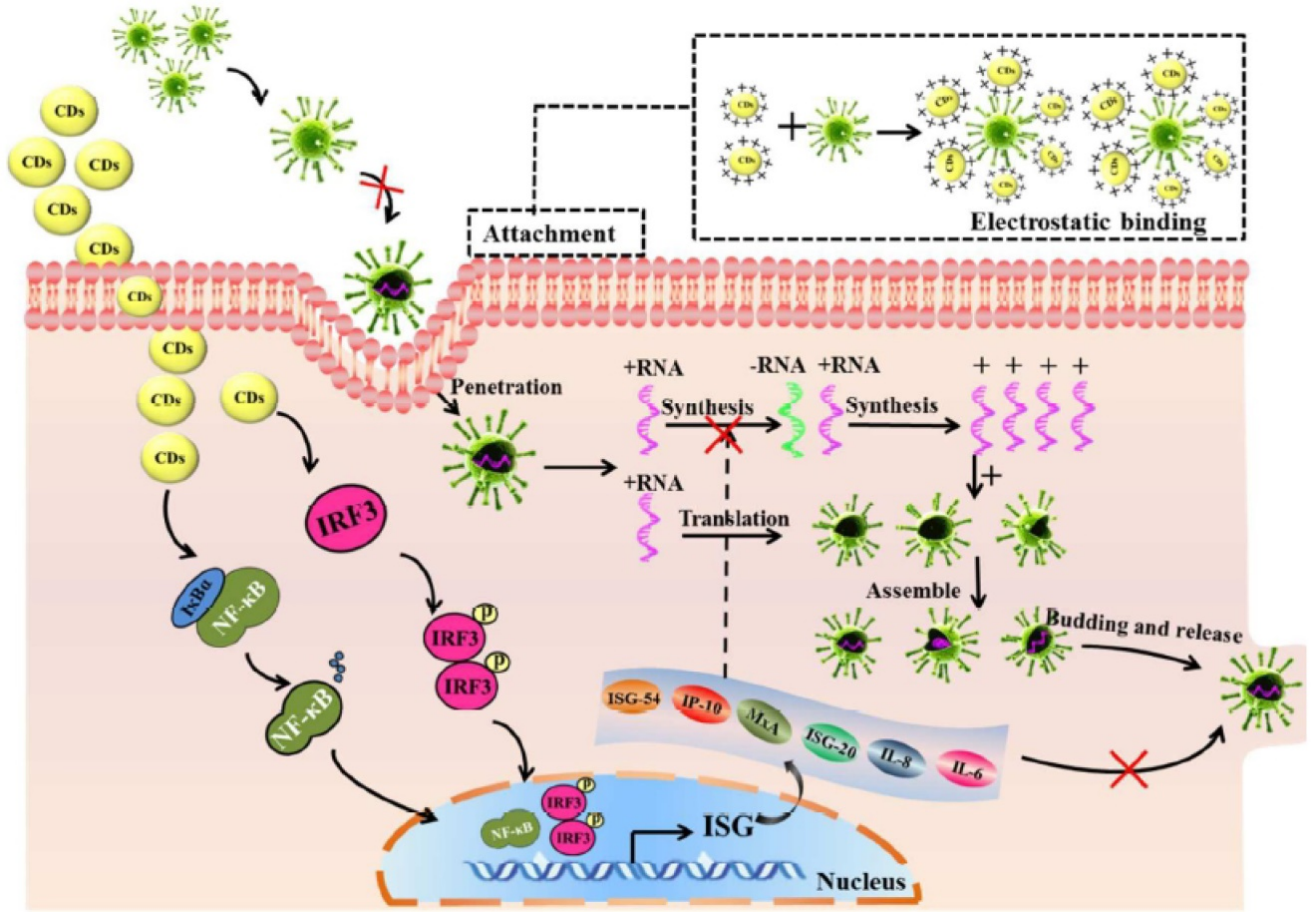
Mechanism of viral inhibition of cationic curcumin-derived CDs. Reprinted with permission from the reference 57.

Furthermore, Ting et al. in 2018 found curcumin-derived CDs could markedly knock down the production of negative RNA strand in porcine epidemic diarrhea virus (PEDV), which was proved by the decrease of negative-strand RNA in curcumin-derived CD-treated Cells in contrast with untreated control at various time intervals after viral infection (Figure 6) 57. The replication of PEDV in Vero cells showed decreasing plaque numbers and reduced virus titers in the CD-treated group compared to the control group 57.

Łoczechin et al. in 2019 prepared functional CDs using ethylenediamine/citric acid as precursors and postmodified the CDs with boronic acid to prevent viral replication of Human Coronavirus 58. The mechanism of action of those CDs was found to be prevention of HCoV-229E entry which was likely due to interaction between surface functional groups of the CDs and HCoV-229E entry receptors; intriguingly, a comparably high inhibition activity of the CDs was evaluated at the viral replication step 58.

**Figure 7:**
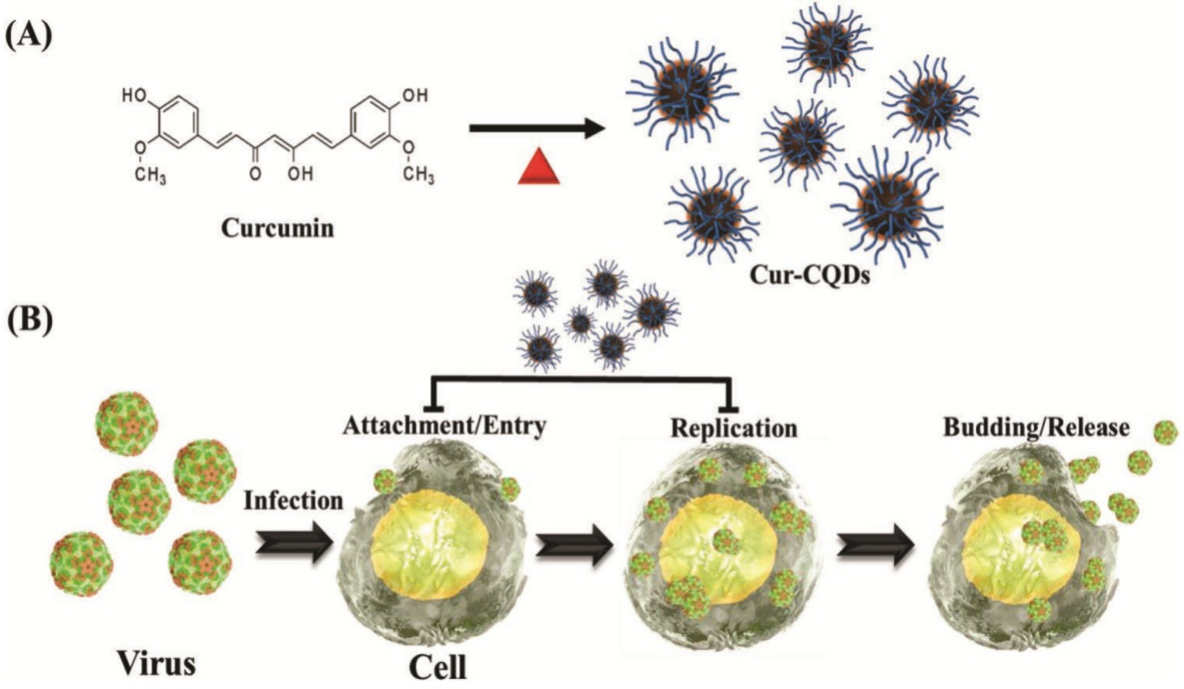
** (A)** The schematic diagram of the one-step synthesis of Cur-CDs. **(B)** The general abstract framework of combating viruses with Cur-CDs. Reprinted with permission from the reference 59.

Lin et al. in 2019 also reported antiviral curcumin based-CDs (Cur-CDs) prepared using a one-step dry-heating handling of curcumin inhibited enterovirus 71 (EV71) as shown in Figure 7. The high biocompatibility of Cur-CDs was demonstrated via *in vitro* cytotoxicity and hemolysis assays. Regulations of synthesis temperature could highly enhance the chemical structural properties of Cur-CDs and their antiviral potency. Their investigation showed that Cur-CDs inhibited EV71 mainly by binding to the virus to prevent viral attachment to the host cell. Furthermore, Cur-CDs could inhibit the viral replication process and prevent the EV71-induced termination of host translation, suggesting that the highly biocompatible Cur-CDs would be a potential agent for therapeutic use against EV71 infection 59.

### Viral Inactivation for Preventing Budding and Detachment Stages

Once the virus replicates, the offspring will bud-off from the host cell as new viruses. The plan of actions which can inhibit the budding and excision of more hazardous viruses can also control the viral infection. Viral contagions can be cured with reactive oxygen species (ROS) that are able to damage DNA via apoptotic controlling signals 57.

CDs-based antiviral agents for preventing virus's budding and detachment were found to be of little information; a few other CDs for anti-viral strategies are only explained to provide more information. Three or four compounds combined to formulating CDs have been observed for a highly antiviral activity against herpes simplex virus (HVS-1). According to the viral protein confirmation, the prepared nanocomposites significantly suppressed the infection of HVS-1 under satisfactorily non cytotoxic concentration. And the mechanism for nanostructures to treat HVS-1 was that they prevented the synthesis of viral negative-strand RNA and viral budding 60.

**Figure 8:**
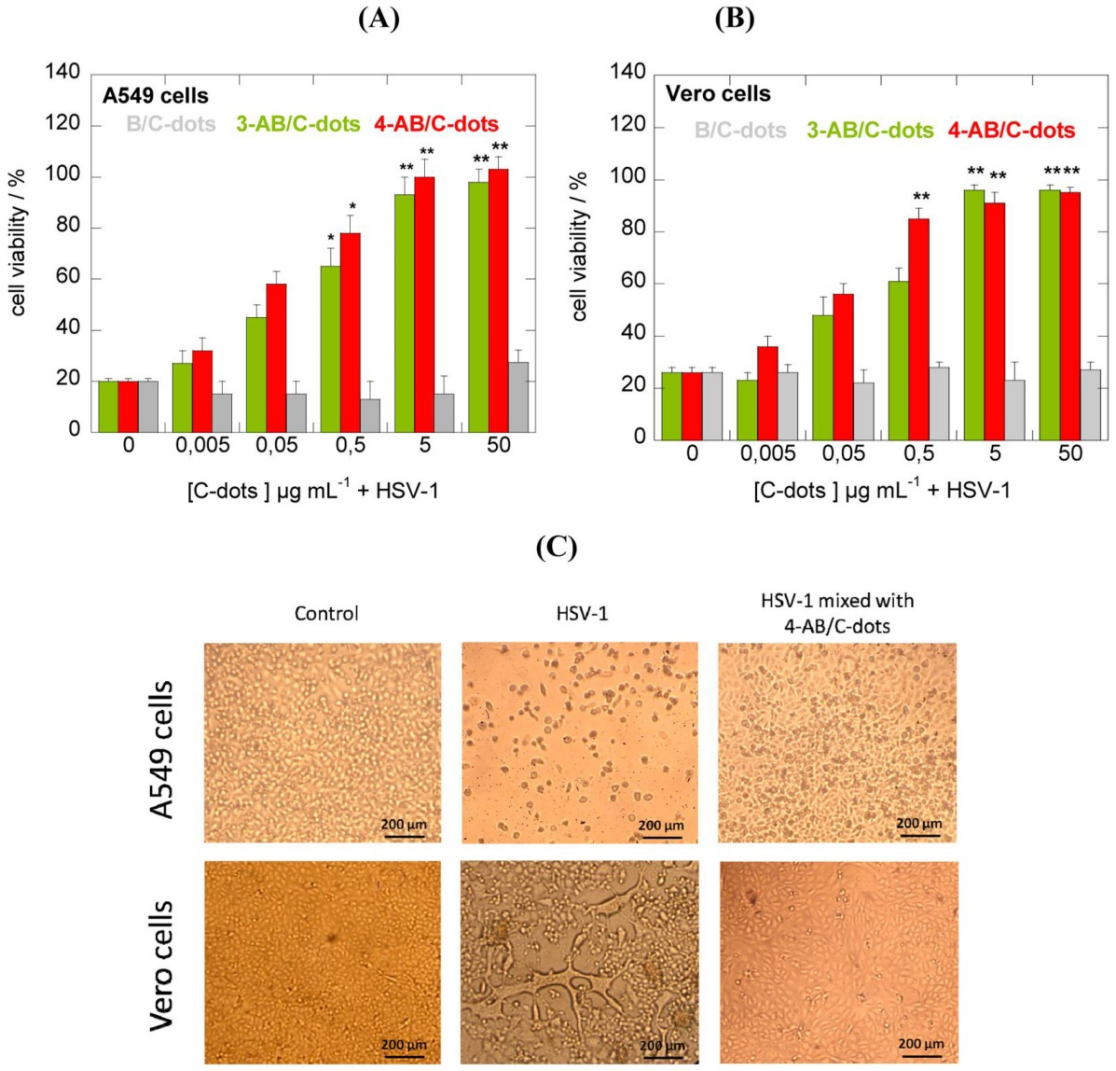
** (A)** CDs inhibition activities of HSV-1 on A549. **(B)** Vero cells at varied concentrations. **(C)** Morphological characteristics of CD effects. Reproduced with permission from the reference 53.

Recent studies have shown the encouraging results to inhibit virus's budding and detachment from the work of nanocomposites modified with boronic acid 61. For example, CDs have been experimented towards *Herpes simplex Virus Type 1* (HSV-1) with potential outcomes *in vitro* studies on monkey kidney cancer cells (Vero) and human lung cancer cells (A549). According to cytotoxicity tests and antiviral assays of formulated CDs from phenylboronic acid (PBA), 3-aminophenylboronic acid (3-APBA), the formation of multiple functional groups of CDs guarantees the effectiveness against virus infection. Interestingly, the role of CDs can be revealed by a morphological study of the cells. Only boronic acid could not inhibit the HSV-1 in comparison to some nanostructures functionalized with boronic acid. Regarding the cytotoxicity study, it was found that prepared CDs were non-cytotoxic towards A549 cells at concentrations of up to 300 μg mL^-1^. As shown in Figure 8, it didn't show no infection, showing 100% cell viability at higher dose than 5 μg mL^-1^ of two prepared 3-APBA/CDs and 4-APBA/CDs 53.

Although three main antiviral mechanisms have been widely found in the use of CDs to treat various viruses, their antiviral activities have also been shown in absence of any presented specific mode of action. Further research is required for investigation of all the plausible modes of strong action of CDs in deactivating, suppressing and killing various RNA viruses.

## Antiviral Activity of Carbon Dots against HIV and other RNA Viruses

As precursors of CDs, some natural products including curcumin, flavonoids and polyphenol compounds showed their intrinsic antiviral, antioxidant, anticancer, anti-inflammatory, and antibacterial functions 59. Ali et al. claimed that curcumin prevents HIV-1 contagion by degrading Tat protein and decreasing the Tat-interposed transcription of the toll-like receptor (TLR) promoter 62. However, they have poor water-solubility in physiological media and low bioavailability *in vivo* systems. So, the use of pure natural products to direct cell lines is limited. In this regard, sustainable, safe, and effective alternatives of these compounds transformation into CDs are urgently required to enhance water-solubility, bioavailability, and antiviral ability due to their unique biological properties, morphological characteristics (e.g. controllable size, structure), and physicochemical characteristics which distinctively differs from those of conventional small-molecule dyes or drugs. Therefore, nontoxic CD-base antiviral agents (non-metallic NPs or sustainable nanomaterials) have highly being recommended owing to their water solubility, biocompatibility or safety, high fluorescence, bioavailability, and synergistic antiviral effect 63, 64. Furthermore, surface modification of CDs has been recently recognized as a cost-effective therapeutic strategy to improve their inhibition towards viral attachment and entry. Ligands present on the surface of CDs could noticeably enhance affinities to multiple cell receptors 53, 58.

Dong et al. in 2017 applied antiviral CDs prepared from different surface passivation molecules, namely 2,2′-(ethylenedioxy)bis(ethylamine) and 3-ethoxypropylamine, to inhibit noroviruses 65. The result showed both CDs significantly inhibited both strains of VLP (virus-like-particles) by binding to histo-blood group antigen (HBGA) receptors on human cells, implying effective inhibition of VLP's binding to HBGA receptors 65.

**Figure 9:**
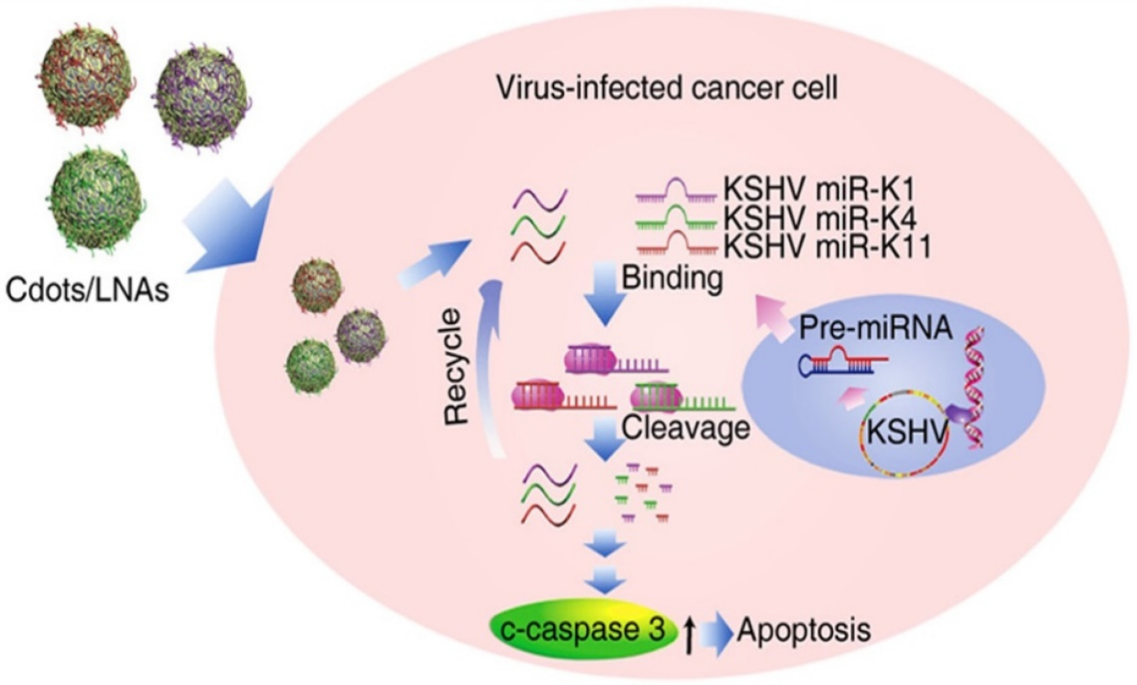
Graphical illustration of viral microRNAs inhibition by CD-interposed drug-loading of LNA for viral therapy. Reproduced with permission from the reference 66.

Ju et al. in 2020 prepared CDs using poly (ethylenimine) and citric acid with microwave-assisted pyrolysis, 66 and the as-prepared CDs were conjugated with locked nucleic acid (LNA) as shown in Figure 9. It was found CD-mediated delivery of locked nucleic acid (CDs-LNA) inhibited the propagation of Kaposi's sarcoma-associated herpesvirus (KSHV) toward initial effusion lymphoma (PEL) cells, inducing apoptosis. The result showed that CDs-LNAs were feasible to deliver model virus suppressors for targeting virus cancers 66.

**Figure 10:**
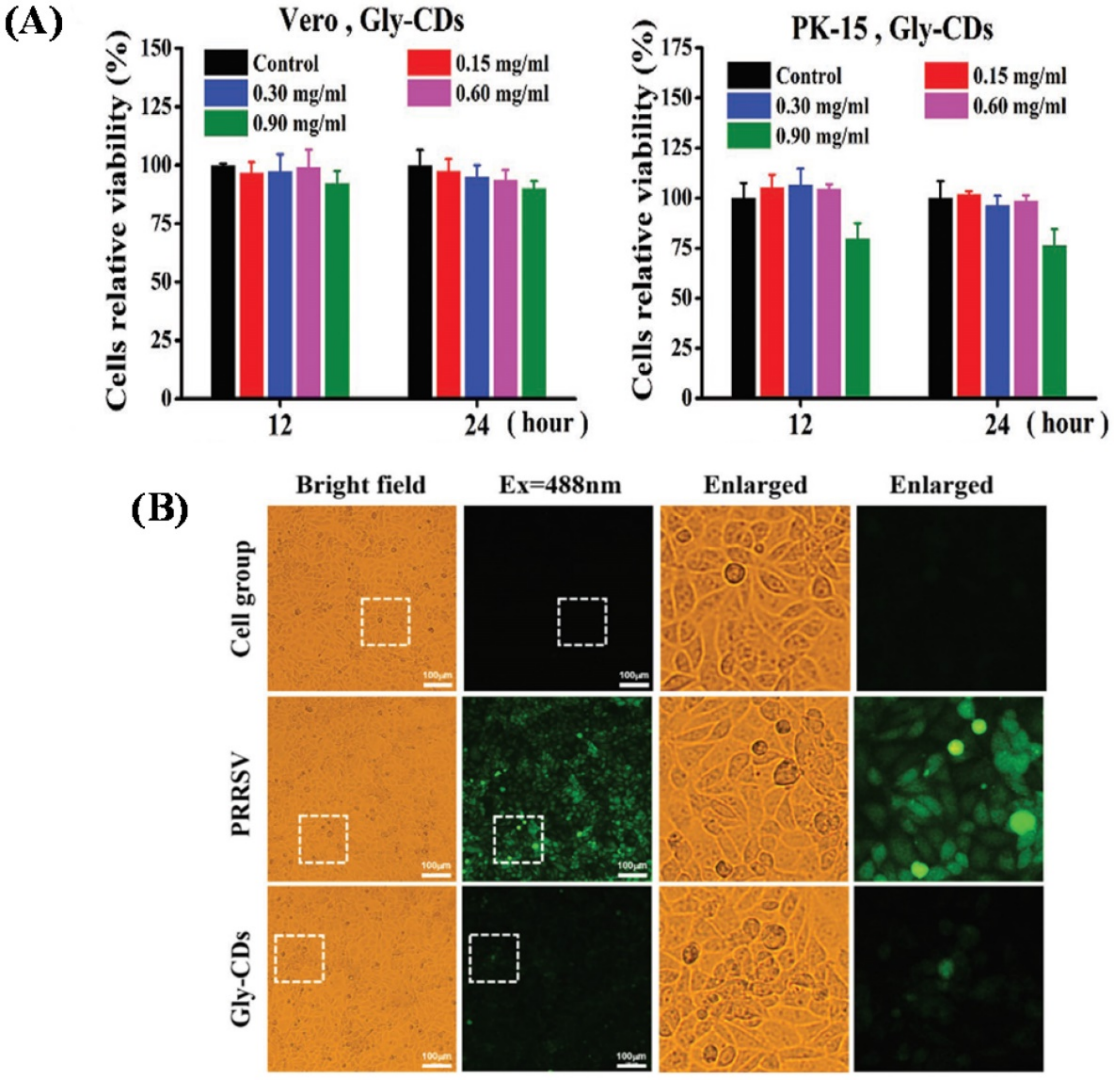
** (A)** The broad-spectrum antiviral efficiency of Gly-CDs. Separate incubation of both Vero and PK-15 cells with Gly-CDs at different concentrations of 0.15, 0.30, 0.60, and 0.90 mg mL^-1^ for 12 and 24 h. (B) Cellular ROS levels in PPRSV infected MARC-145 cells post different treatments. The cell group shows normal cells not treated by either Gly-CDs or PRRSV. The mock group exhibits the PRRSV-infected cells in the absence of Gly-CDs. The Gly-CDs group indicates the PRRSV-infected cells treated with Gly-CDs. Scale bar = 100 µm. Reprinted with permission from the reference 67.

Tong et al. in 2020 prepared glycyrrhizin acid-based CDs (Gly-CDs) from Chinese herbal medicine via hydrothermal treatment. The result indicated that Gly‐CDs could inhibit the porcine propagation and respiratory syndrome virus (PPRSV) invasion and reproduction by stimulating antiviral innate immune systems. The viabilities of both Vero and PK cells make it possible to apply them in biomedical fields considering that the mean percentage of their proliferation rate is above 80% as depicted in Figure 10A. Gly-CDs also inhibited the aggregation of intracellular ROS affected by PRRSV infection as indicated in Figure 10B so they were considered as a potential therapeutic agent for alternative theranostics of PRRSV contagion 67.

Moreover, the use of graphene quantum dots (GQDs) has to be mentioned as an antiviral strategy to harmonize CDs positioning on virus inhibition since GQDs are one of family members of CDs and both of them share multiple superior virtues. CDs stand for an extensive group of carbon-based nanoparticles, their structures are still far from being fully understood. Typically, carbon-dominated nanomaterials are CDs, amorphous carbon nanoparticles, partially graphitized core-shell carbon nanoparticles, amorphous fluorescent polymeric nanoparticles and graphene quantum dots (GQDs). All of them belong to carbon-based fluorescent nanomaterials but their sizes are different 68. Many graphene lattices which are found in the inner part of GQDs are generated from graphene-based precursors or the rigid synthetic process with graphene-like polycyclic hydrocarbon molecules 69. Recently, reduced graphene oxide (rGO) has been employed for label-free detection of influenza viruses due to its promising conducting properties and large surface area 70. For example, the interaction of rGO sulphated derivatives and viruses exhibits the degree of sulfation and polymer density-dependent 71. The stronger the level of sulfation and the smaller the size, the more powerful effect was observed on herpes virus. It was implied as a combination outcome of the facile bending and shared encapsulation by two or more small GO sheets 71. In fact, GO scales can wrap up and impound microorganisms by enveloping them in a covering carbon sheet 72. Interestingly, it has been lately explored on how the SARS-CoV-2 protein-receptor-binding domain could correlate with heparin that in turn alters conformation, which demonstrates how to develop a first-line therapeutic agent against heparin viruses with glycosaminoglycan-based antiviral drugs kin the presence of sulphated derivatives of GO. Graphene is also used for biosensing and bioimaging since photocatalyzed graphene-derived light absorption can be utilized to knock down viral particles. Also, sulfonated magnetic NPs which were functionalized with rGO have been used to effectively track and photothermally diminish herpes simplex virus type 1 (HSV-1) using NIR light 73, 74.

Xiang et al. in 2018 prepared a simple and smart DNA detection probe from GQDs-modified glassy carbon electrode combined with specific sequence DNA molecules to identify hepatitis B Virus (HBV)'s DNA 75. The probe DNA would adhere to the target, HBV-DNA, instead of GQDs if the target HBV-DNA was found in the test solution. Also, the study suggested that the sensor could be used in detecting other probe DNA with high sensitivity 75.

**Figure 11:**
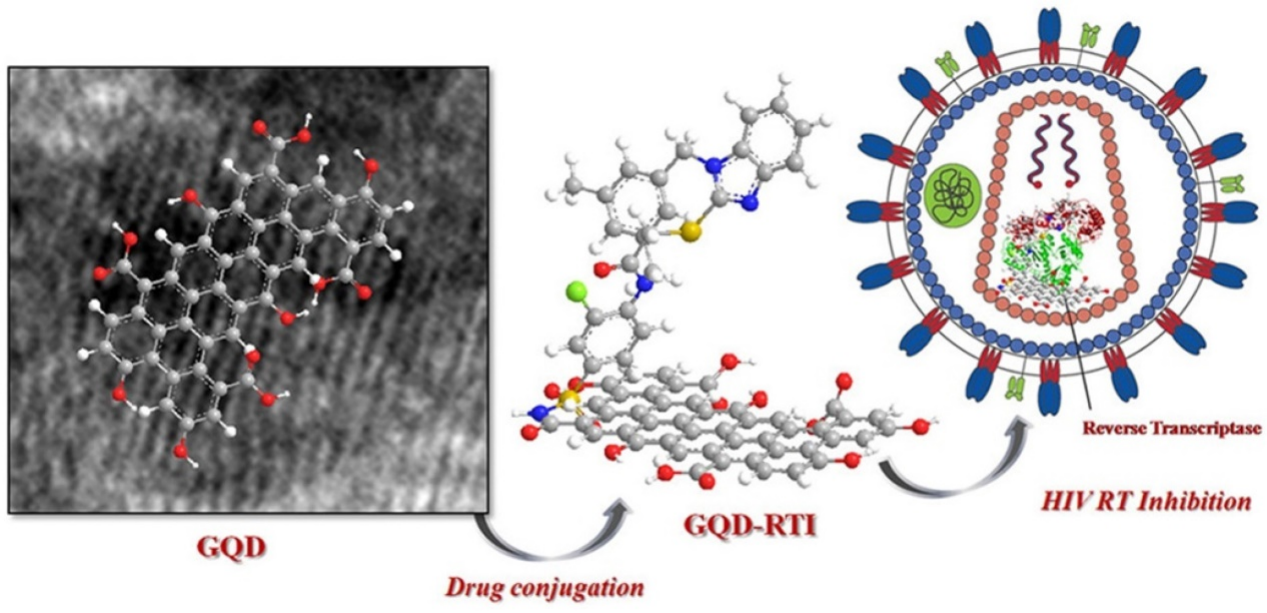
The reverse transcriptase inhibitor-conjugated GQDs as a promising therapeutic candidate for HIV treatment. Reprinted with permission from the reference 76.

Iannazzo et al. in 2018 reported a type of GQDs prepared from multiwalled carbon nanotubes (MWCNTs) using lengthy acidic oxidation and exfoliation process 76. The synthesized GQDs were conjugated with the reverse transcriptase inhibitors CHI499 and CDF119 to be retroviral agents as indicated in Figure 11. As a result, the prepared modified GQDs inhibited viral entry of HIV with non-toxicity. Therefore, it is conferred from their promising results that a novel antiviral strategy using GQDs could be explored for HIV inhibition and therapy 76.

The published researches on CDs and other NPs for antiviral activities are summarized in the Table 1.

The collected articles have proven the great efficiency of CD-based antiviral agents derived from molecules, compounds, and combining with complex nano-substances. And promising antiviral results from some published articles of facile prepared-CDs and complex mixed-compounds by surface modifications or conjugation are demonstrated. The above-mentioned articles mostly focused on virus inhibition and the life cycle of viruses affected by the antiviral activity from one or more CD species. In addition, it is clear that CD-based antiviral agents prepared from natural curcumin also exhibited antiviral effects on RNA viruses. Besides, it is noticeable that surface modifications and functionalization of CDs have a highly strong preference in enhancing the efficiency of CD-based antiviral agents.

## Photodynamic Inactivation

Photosensitized carbon-based nanomaterials are also needed as sensitive, selective, and affordable biosensors for tracking and killing viruses in this pandemic situation. Henceforth, CDs have been explored in viral as well as bacterial sensing or killing. Biosafety of CDs can influence the diagnosis and management of various viral diseases. The affordable fluorescence feature can make CDs as sensors or photosensitizers for biological as well as non-biological operations 81. For instance, photodynamic inactivation (PDI) derived from CDs has been used to inactivate microorganisms 82.

In PDI, a well-known method is to use UV light to illuminate on focused NPs such as colloidal TiO_2_ to produce ROS, including singlet oxygen, superoxide and hydroxyl radicals, to kill pathogenic bacteria or viruses. Since UV light is harmful for extensive uses in human, TiO_2_ NPs have been modified to extend absorption to visible length. Upon photoexcitation of CDs, electrons and holes are excited, which are trapped at various defect sites in the dots and might directly take part in the explored antimicrobial activities. Subsequently, the electrons and holes after recombination result in the PL emission of CDs, which also participates in the photodynamic antimicrobial functions 83.

Lim et al. in 2012 presented up-conversion NP-based photodynamic inactivation for potential antiviral uses 84. Those up-conversion NPs acted as photosensitizers in target biological environment converting NIR to visible emission. Interestingly, the up-conversion NPs significantly reduced the infectious rate of viruses *in vitro* after photodynamic treatment 84.

**Figure 12:**
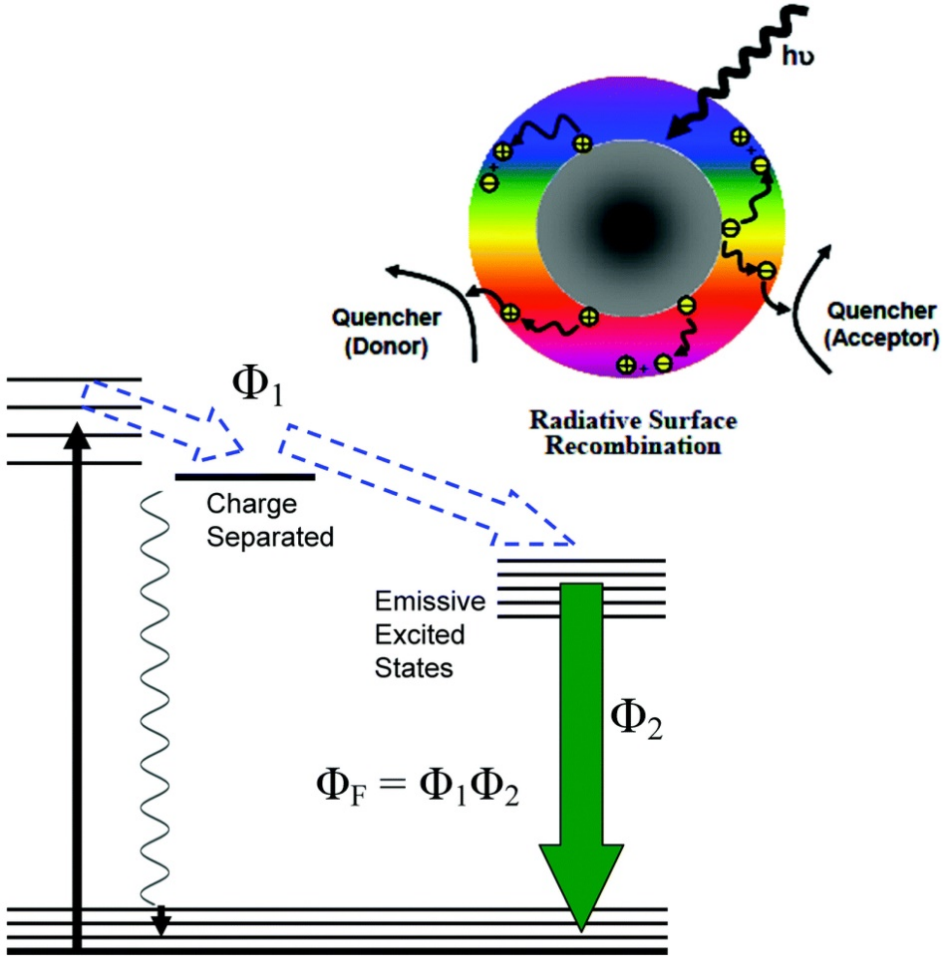
Above: A schematic description of CDs on the photoexcited state processes, separating charge, trapping electrons, and creating holes and their radiative recombinations. Below: The energy transformation process with the obtained fluorescence quantum yield (ΦF). Reprinted with permission from the reference 83.

Rabe et al. in 2020 designed a type of CDs coupled with oligometric polyethylenimne 83. The synthesized CDs exhibited visible emission, which readily inactivated multidrug-resistant bacterial strains as depicted in Figure 12 83. Highly photosensitized CDs were observed under nature light exposure to inactivate small RNA viruses of MS2 by degrading the viral genomic RNA. However, the prepared CDs showed that no significant damage to the MS2 phage shell 85.

Action and outcome of photoexcited CDs contribute to the PDI capability in highly visible/natural light-activated antibacterial function of CDs. To show more evidences of clear understanding in mechanistic details, CDs need to be further studied for broad applications in the prevention and control of viral infection/spread.

## Conclusion and perspectives

In summary, CDs and GQDs were revealed as promising antiviral agents from many studies on HIV or other RNA viruses' inhibition. Various approaches for the preparation of CDs have highlighted their advantages over virus inhibition and treatment *in vitro*, but a few challenges such as practical testing *in vivo* systems is still a matter of time. Also, targeting and long-lasting of CDs in virus-infected cells could be significantly enhanced only when their surfaces were coated with diverse ligands. In contrast, although GQDs, GO and graphene-based nanomaterials considerably displayed strong capabilities to detect viruses, their sizes are larger than that of CDs. Subsequently, GQDs are less preferable than CDs since the direct use *in vivo* could not have controllable and predictable antiviral loading capabilities. In the viewpoint of photodynamic therapy, the responsive polymer materials can be used to prepare NPs, which can respond to enzymes, ROS, or other chemicals abundant in the infection sites to overcome the uncontrolled inflammation and release of cytokines. Nevertheless, it is hoped that aforementioned results encourage the development of CDs and GQDs as antiviral agents for new generations to treat virus infections.

## Figures and Tables

**Figure 1:**
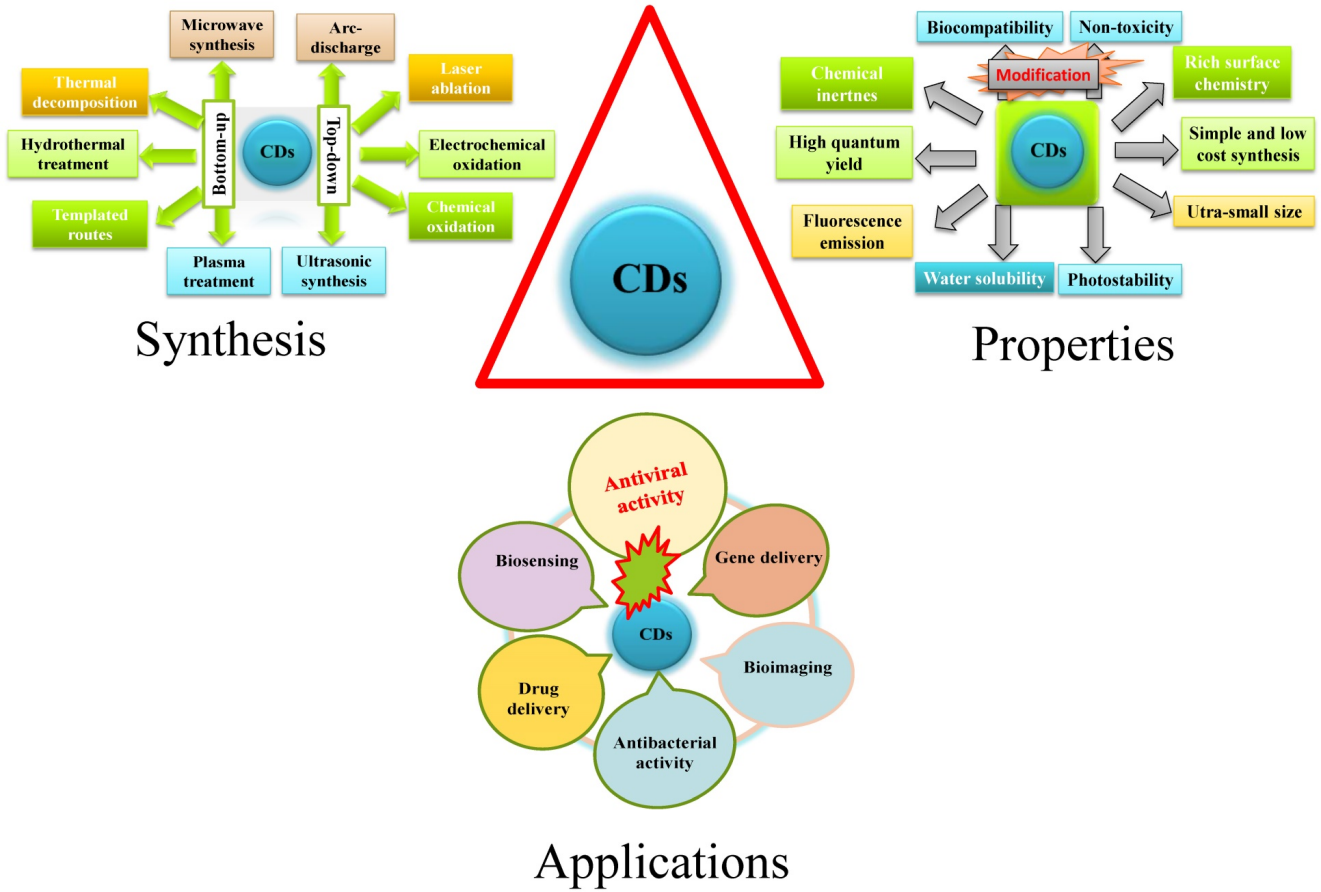
A general schematic diagram of synthesis, properties, and applications of CDs.

**Table 1:** Carbon dots or other NPs developed for antiviral activities

CDs	Precursors/Routes	Size, morphology, toxicity, and IC50	Cells/Virus	Purpose	Reference
PBA-CDs 3-APBA-CDs 4-APBA-CDs	Boronic acid/ Hydrothermal carbonization	•Size diameters = 332±60 nm, 55 ±1nm, 96 ± 1nm, •Cell proliferation = 67%, 95%, and 97% •IC50 = 80 and 145 mg/mL^-1^	•A549 cells & Vero cells/ •HSV-1	•For stopping viral attachment and entry.	53
CCM-CDs	Citric acid/ Pyrolysis	•Diameter = 1.5 ± 0.3 nm •Cell proliferation = 90%	•Vero cells/ •Coronavirus	•For inhibiting viral entry •In order to prevent budding of negative-strand RNA in virus.	77
Young barley leaf-derived B-CDs	Citric acid/ Hydrothermal	•Size in diameter = 1.9 nm •Cell survival ≥ over 85%	•Hela cells & PK-15 cells/ •Pseudorabies virus (PRV)	•For evaluating bioimaging and antiviral effects	78
Citric acid modified by boronic acid CDs	Carboxyl phenylboronic acid (CBBA)/ Pyrolysis	•Diameter = 2.8 nm - 6.2 nm •Cell proliferation < 80% •IC50 = 9506.3 and 26.7 μg/mL	•MT4 and MOLT-4 cells/ •HIV-1	•For inhibiting HIV infection	26
BZM-CDs	Hydrothermal	•Average size = 4.4 ± 0.6 nm •Cell survival rate >80%	•BHK-21 cells & Vero cells/ •Flaviviruses	•For blocking the viral infection	54
Ethylenediamine + citric acid (CQDs)	Boronic acid/ Hydrothermal carbonization	•Particle size = 6.5 ± 0.2 nm •EC50 = 52 ± 8 μg/ mL	•Huh-7 cells/ •HCoV	•For inhibiting viral replication.	58
Citric acid and ethylene diamine (CQDs)	Streptavidin/ Hydrothermal	•Particle size = 4 - 5nm	•HIV-1	•For assessing HIV-1 p24 antigen on improved models	79
Cur-CQDs	Pyrolysis	•Mean diameter = 4.8 ± 0.8 nm •CC50 = 452.2 µg/ mL •EC50= 0.2 µg/ mL	•RD cells/ •EV71	•For inhibiting EV71 infection	59
Spermidine powder -based CDs	Biogenic polyamines/ Pyrolysis	•Size = 6 nm	•Haemolymph of shrimp *Litopenaeus vannamei* •White spot syndrome virus (WSSV)	For blocking receptor on host cell membrane using recombinant viral proteins or virus antiserum	80

## Abbreviations

NPs: nanoparticles
CDs: carbon dots
PL: photoluminescence
PBA: 4-phenylboronic acid
3-APBA: 3-aminophenylboronic acid
4-APBA: 4-aminophenylboronic acid hydrochloride
PBMCs: peripheral blood mononuclear cells
CCM-CQDs: cationic curcumin-based carbon quantum dots
BZM-CQDs: benzoxamine-based carbon quantum dots
Cur-CQDs: curcumin-based carbon quantum dots
GO: graphene oxide
GQDs: graphene quantum dots
rGO: reduced graphene oxide
